# Relationship between obesity, ethnicity and risk of late stillbirth: a case control study

**DOI:** 10.1186/1471-2393-11-3

**Published:** 2011-01-12

**Authors:** Tomasina Stacey, John MD Thompson, Edwin A Mitchell, Alec J Ekeroma, Jane M Zuccollo, Lesley ME McCowan

**Affiliations:** 1 Department of Obstetrics and Gynaecology, University of Auckland, ACH Support Building, Park Road, Grafton, Auckland 1020, New Zealand; 2 Department of Health Sciences, AUT University, Akoranga, Auckland, New Zealand; 3 Department of Paediatrics, University of Auckland, ACH Support Building, Park Road, Grafton, Auckland 1020, New Zealand; 4 Department of Obstetrics and Gynaecology, Wellington Medical School, Wellington, New Zealand

## Abstract

**Background:**

In high income countries there has been little improvement in stillbirth rates over the past two decades. Previous studies have indicated an ethnic disparity in the rate of stillbirths. This study aimed to determine whether maternal ethnicity is independently associated with late stillbirth in New Zealand.

**Methods:**

Cases were women with a singleton, late stillbirth (≥28 weeks' gestation) without congenital abnormality, born between July 2006 and June 2009 in Auckland, New Zealand. Two controls with ongoing pregnancies were randomly selected at the same gestation at which the stillbirth occurred. Women were interviewed in the first few weeks following stillbirth, or at the equivalent gestation for controls. Detailed demographic data were recorded. The study was powered to detect an odds ratio of 2, with a power of 80% at the 5% level of significance, given a prevalence of the risk factor of 20%. A multivariable regression model was developed which adjusted for known risk factors for stillbirth, as well as significant risk factors identified in the current study, and adjusted odds ratios and 95% confidence intervals were calculated.

**Results:**

155/215 (72%) cases and 310/429 (72%) controls consented. Pacific ethnicity, overweight and obesity, grandmultiparity, not being married, not being in paid work, social deprivation, exposure to tobacco smoke and use of recreational drugs were associated with an increased risk of late stillbirth in univariable analysis. Maternal overweight and obesity, nulliparity, grandmultiparity, not being married and not being in paid work were independently associated with late stillbirth in multivariable analysis, whereas Pacific ethnicity was no longer significant (adjusted Odds Ratio 0.99; 0.51-1.91).

**Conclusions:**

Pacific ethnicity was not found to be an independent risk factor for late stillbirth in this New Zealand study. The disparity in stillbirth rates between Pacific and European women can be attributed to confounding factors such as maternal obesity and high parity.

## Background

Stillbirth remains a common and devastating pregnancy complication. Approximately 8 in 1000 New Zealand births result in stillbirth (fetal death ≥ 20 weeks gestation) and more than 1 in 300 result in late stillbirth (death ≥ 28 weeks gestation)[1]. Unfortunately the decline in stillbirth rate in New Zealand and elsewhere that occurred in the 1980's and 1990's has not been sustained in the last decade [1-4].

Previous retrospective New Zealand studies have documented ethnic disparity in the rate of stillbirth, with Pacific women having an increased risk of stillbirth compared to European women [5,6]. Ethnic disparity in stillbirth risk has also been reported elsewhere. In the UK, both Black and Asian women have a disproportionate number of stillbirths compared to White women [4,7,8]. In the United States, African American women have more than twice the risk of stillbirth compared to White women [9,10]. The reasons for these reported ethnic disparities have not yet been determined.

International studies have reported several demographic risk factors associated with stillbirth, including; advanced maternal age [11,12], obesity [13,14] smoking in pregnancy [15], low socio-economic status [16] and extremes of parity [17,18]. Many previous studies that have reported ethnic disparity in the risk of stillbirth were retrospective in design and had insufficient variables to enable appropriate adjustment for confounders [6,7,9].

The main aim of the prospective Auckland Stillbirth Study was to identify modifiable risk factors for late stillbirth. We hypothesised that, after adjustment for demographic risk factors, the risk of late stillbirth amongst Pacific women would not differ from that in European women and that obesity would be an independent risk factor for late stillbirth.

## Methods

All women booked to give birth in the greater Auckland region and who experienced a stillbirth at or after 28 weeks gestation between July 2006 and June 2009 were eligible to participate in the study. Late stillbirth was selected as the primary outcome as live born babies born at or after 28 weeks are likely to survive. Women with multiple pregnancies and those where the baby died due to a congenital abnormality were excluded. Each case was matched with two randomly selected controls with an ongoing pregnancy at the same gestation at which the stillbirth occurred. A description of the manner in which cases and controls were identified and recruited, and other detailed methods have been described previously [19]. Data were obtained through interviewer administered questionnaires and from clinical data extraction.

Ethical approval was gained for this study from the Northern "X" Regional Ethics Committee.

### Data collection

Interviews took place in the first few weeks following the stillbirth, or for controls, at the equivalent gestation of pregnancy at which the stillbirth occurred. Demographic data included maternal age, ethnicity and country of birth, body mass index, education level, social deprivation index, work status, marital status, parity, smoking and recreational drug use during pregnancy.

A single ethnicity was self assigned using a system of prioritisation based on the standardised system of ethnicity data collection used by the New Zealand Ministry of Health [20]. In this study, due to small numbers, all non-Maori, non-Pacific, non-European groups were combined and defined as 'other'.

Maternal Body Mass Index (BMI) was calculated from the earliest known weight taken in pregnancy and maternal height measured at interview. BMI was considered as a continuous, and also as a categorical, variable. BMI was classified according to two criteria: conventional World Health Organisation (WHO) [21] and ethnic specific [22]. Only 2 (1.3%) cases and 6 (1.9%) controls were classified as underweight, and BMI was therefore categorised into 3 groups: <25 kg/m^2^, 25-29.9 kg/m^2^, and ≥ 30 kg/m^2 ^for WHO criteria. Appropriate cut off points for overweight and obesity differ between ethnic groups as the BMI and body fat ratios differ [23,24] and consequently ethnic specific categories have been developed for use in multi ethnic populations [25]. For Asians, overweight was defined as ≥23 kg/m^2 ^and obesity as ≥27.5 kg/m^2 ^and for Pacific/Maori, overweight is ≥26 kg/m^2 ^and obesity >32 kg/m [21,22]. Three ethnic specific BMI categories were therefore also created: normal/underweight, overweight and obese. As ethnic specific BMI categories are not universally accepted, the conventional WHO criteria were used in the multivariable analysis.

The level of social deprivation (Deprivation Index) was determined by the address at which the participant was living at the time of stillbirth/interview, based on the 2006 New Zealand Deprivation Index (NZDep2006) [26]. Smoking status was assigned to one of three groups: smoking at any time during pregnancy (smoker), non smoker who lived with a smoker (passive only), and those that did not smoke in pregnancy and lived in a smoke free environment (smoke free).

### Analysis

The study was powered to detect an odds ratio of 2, with a power of 80% at the 5% level of significance, given a prevalence of the risk factor of 20%. All statistical tests were performed using SAS (SAS Institute Inc., Cary NC USA). Standard conditional regressions were used for matched case control studies using the 'proc logistic' procedure, with the 'strata' statement to control for matching. Continuous variables were compared using t-tests. Statistical significance in multivariable analysis was defined at the 5% level. Odds ratios (OR) and adjusted odds ratios (aOR) with 95% confidence intervals were used to calculate risk in univariable and multivariable analysis respectively.

The main multivariable regression model included maternal variables known to be associated with increased risk of stillbirth, based on evidence from previous literature (maternal age, BMI, ethnicity, parity, smoking and socio-economic status). Other demographic variables from this study with a p-value of <0.10 were also included in the model.

## Results

During the study period 215 eligible cases of late stillbirth were identified, 155 (72%) consented to take part in the study, as did 310/429 (72%) of the eligible controls. No significant difference was identified between the characteristics of those who consented and those who did not [19].

In univariable analysis, Pacific women were found to have an increased risk of late stillbirth compared to European women (OR 1.88 95%CI 1.13-3.13), whereas the risk for Maori women did not differ [Table 1]. There was no significant difference in the gestation at which the earliest pregnancy weight was measured (14.3 weeks (SD 7.0) for cases and 13.8 weeks (SD 7.1) for controls *p *= 0.54). Using conventional WHO BMI groupings, both overweight and obese women were found to have an increased risk of stillbirth relative to normal/underweight women. A similar pattern, with slightly reduced odds ratios, was also found when ethnic specific BMI criteria were used.

**Table 1:** Characteristics of women with late stillbirth compared with gestation matched controls

	Cases		Controls		Univariable OR 95% CI	
	n = 155	(%)	n = 310	(%)		
***Maternal age***						
*<20*	10	(6.5)	24	(7.7)	0.80	0.38-1.71
*20-34*	113	(72.9)	216	(69.7)	1.00	-
≥ *35*	32	(20.7)	70	(22.6)	0.86	0.53-1.42

***Maternal ethnicity***						
*Maori*	19	(12.3)	46	(14.8)	1.08	0.57-2.04
*Pacific*	48	(31.0)	67	(21.6)	1.88	1.13-3.13
*European*	55	(35.5)	139	(44.8)	1.00	-
*Other*	33	(21.3)	58	(18.7)	1.46	0.85-2.51

***Country of birth***						
*New Zealand*	83	(53.6)	168	(54.2)	1.00	-
*Other*	72	(46.5)	142	(45.8)	1.03	0.69-1.54

***Marital status***						
*Married*	84	(54.2)	202	(65.2)	1.00	-
*Not married*	71	(45.8)	108	(34.8)	1.60	1.07-2.38

***Deprivation Index***						
*1-4*	91	(58.7)	218	(70.3)	1.00	-
*5 (most deprived)*	64	(41.3)	92	(29.7)	1.74	1.14-2.67

***School leaving age***						
*<17 years*	36	(23.2)	56	(18.1)	1.37	0.85-2.18
≥*17*	119	(76.8)	254	(81.9)	1.00	-

***Employment in last month***						
*Paid work*	41	(26.5)	118	(38.1)	1.00	-
*Not in paid work*	114	(73.6)	192	(61.9)	1.75	1.13-2.70

***Parity***						
*0*	77	(49.7)	144	(46.5)	1.41	0.94-2.13
*1-3*	62	(40.0)	156	(50.9)	1.00	-
≥*4*	16	(10.3)	10	(3.2)	5.13	1.99-13.21

***BMI***						
*< 25*	55	(35.5)	156	(50.3)	1.00	-
*25-29.9*	39	(25.2)	67	(21.6)	1.69	1.03-2.78
≥ *30*	61	(39.4)	87	(28.1)	2.08	1.30-3.33

***Ethnic specific BMI groups***						
*Normal/underweight*	57	(36.8)	151	(48.7)	1.00	-
*Overweight*	44	(28.4)	81	(26.1)	1.46	0.91-2.35
*Obese*	54	(34.8)	78	(25.2)	1.91	1.18-3.09

***Smoking in pregnancy***						
*Smoke-free*	80	(51.6)	201	(64.8)	1.00	
*Passive only*	29	(18.7)	43	(13.9)	1.67	0.99 - 2.83
*Smoker*	46	(29.7)	66	(21.3)	1.78	1.11 - 2.85

***Recreational drugs***						
*No drug use*	142	(91.6)	298	(96.1)	1.00	-
*Any drug use*	13	(8.4)	12	(3.9)	2.35	1.02 - 5.42

***Previous preterm birth***						
*Yes*	11	(7.10)	15	(4.84)	1.52	0.67 - 3.43
*No*	144	(92.80)	295	(95.16)	1.00	-

***Previous small for gestational age***						
*Yes*	12	(7.74)	15	(4.84)	1.60	0.75 - 3.42
*No*	143	(92.26)	295	(95.16)	1.00	-

***Previous stillbirth***						
*Yes*	2	(1.29)	3	(0.97)	1.33	0.22 - 7.98
*No*	152	(98.71)	307	(99.03)	1.00	-

Women who were; grandmultiparous (parity ≥4), not married, lived in the most deprived areas, or were not in paid work were also found to be at increased risk of late stillbirth. Both cigarette smokers and non smokers regularly exposed to environmental cigarette smoke had a similar degree of increased risk. Recreational drug use was associated with a greater than twofold increase in risk. Marijuana was the most commonly used recreational drug in pregnancy in 9 (5.8%) cases and 11 (3.6%) controls, and the majority of marijuana smokers also smoked cigarettes; 82% of marijuana smoking cases and 100% of marijuana smoking controls also smoked cigarettes.

In this study, no significant relationship was found between risk of late stillbirth and maternal age, country of birth or school leaving age. Previous adverse pregnancy outcome was not found to be significantly associated with late stillbirth risk in this study.

After multiple regression analysis was performed, adjusting for potential confounders, maternal Pacific ethnicity was no longer found to be independently associated with late stillbirth (aOR 0.99 95%CI 0.51-1.91), and Maori ethnicity was now found to be associated with a significantly reduced risk of late stillbirth (aOR 0.41 95%CI 0.17-0.96) [Table 2]. Maternal overweight and obesity remained significant, with obese women having a more than 2 fold increase in risk compared to normal weight/underweight women (aOR 2.11; 95% CI 1.14-3.91). As obesity is known to be associated with pre-eclampsia and diabetes, we fitted an additional model including these two variables (prevalence 0.9% and 4.5% respectively). The findings of the original model were unchanged when adjustment was made for these additional variables.

**Table 2:** Multivariable odds ratios for maternal characteristics associated with late stillbirth

	Adjusted OR* (95% CI)	
***Maternal age***		
*<20*	0.61	0.24-1.52
*20-34*	1.00	-
≥*35*	0.82	0.46-1.46

***Maternal ethnicity***		
*Maori*	0.41	0.17-0.96
*Pacific*	0.99	0.51-1.91
*European*	1.00	-
*Other*	1.69	0.92-3.11

***Marital status***		
*Married*	1.00	-
*Not married*	1.75	1.03-2.98

***Deprivation Index***		
*1-4*	1.00	-
*5*	1.20	0.72-2.00

***Employment in last month***		
*Paid work*	1.00	-
*Not in paid work*	1.66	1.00-2.77

***Parity***		
*0*	1.75	1.08-2.83
*1-3*	1.00	-
≥*4*	4.22	1.44-12.40

***BMI***		
*< 25*	1.00	-
*25-29.9*	1.75	1.00-3.05
≥ *30*	2.11	1.14-3.91

***Smoking***		
*Smoke-free*	1.00	-
*Passive only*	1.36	0.74-2.47
*Smoker*	1.28	0.71-2.32

***Recreational drugs***		
*No drug use*	1.00	-
*Any drug use*	2.77	0.96-7.99

## Discussion

Auckland, the largest city in New Zealand, contains the greatest numbers of Pacific people of any city in the world [27]. The increased rate of late stillbirth amongst Pacific women compared to European found in univariable analysis is consistent with previous studies from New Zealand and with other international studies indicating ethnic disparity in stillbirth risk [4,9,28,29]. This study was able to adjust for a number of potential demographic confounders such as obesity, parity and poverty. After this adjustment, Pacific ethnicity was no longer found to be independently associated with an increased risk of late stillbirth.

The results from this study also suggest that after adjusting for known confounding factors, Maori ethnicity was associated with a reduced risk of late stillbirth. This finding seems inconsistent with two other studies which reported that Maori women had a similar overall stillbirth risk to European women [6,28], although these former studies were not able to adjust for BMI, smoking or drug use in pregnancy. There is also evidence that Maori women may have an increased risk of stillbirth prior to 28 weeks gestation, rather than late stillbirth [30]. Our findings of a reduced rate of late stillbirth in Maori, when considered with other New Zealand data suggesting an increase in early stillbirth, are compatible with the previous studies which showed no overall increase in stillbirth risk for Maori compared with European [6,28]. However our data suggest that Maori women who do not have lifestyle risk factors may have a reduced risk of late stillbirth, and future well designed studies are needed to confirm or refute these findings.

Consistent with previous research, we found a twofold increase in late stillbirth risk in obese women [13,14,31,32]. Obesity is particularly prevalent amongst Pacific women in New Zealand, with almost 64% of all Pacific women being classified as obese [33]. Obesity is also more prevalent in African Americans compared to White women in the United States [34]. Few previous studies showing disparity in stillbirth risk between ethnic groups have been able to adjust for BMI [9,28]. One study, by Salihu and others, which has explored this relationship, reported that obesity compounded the stillbirth risk for African American women, as the increased risk of stillbirth for obese African American women was on average 50% greater than that for obese White women [32].

A novel feature of this study is that we have investigated the risk of stillbirth using ethnic specific as well as conventional WHO BMI criteria. The relationship between obesity and stillbirth was similar regardless of which criteria were used.

The mechanism(s) by which obesity increases the risk of stillbirth is not clear and is likely multifactorial. Metabolic disorders such as diabetes and pre-eclampsia are associated with obesity [35], and are in turn associated with an increased risk of stillbirth [36]; however in our study adjustment for these factors did not diminish the independent effect of obesity. Obese pregnant women have altered metabolic profiles compared with non obese women, including higher glucose levels (even when not meeting criteria for diabetes), altered lipids and a pro-inflammatory milieu [37]. In high income countries, obesity is associated with lower socio-economic status, and women who do not have access to micronutrient rich food have been found to be more likely to be overweight or obese [38]. In this study, however, adjustment for socio-economic status did not reduce the effect of obesity on the risk of stillbirth. Further research is required to determine whether nutritional status has an impact on the risk of stillbirth [39]. Obese pregnant women have significantly more sleep-related disordered breathing than normal weight women [40]. Snoring has been associated with fetal growth restriction and pregnancy-induced hypertension [41], but there has been only one case study reported that suggested a link between obstructive sleep apnoea and stillbirth [42]. Obesity has also been associated with altered perception of fetal movements, with more overweight and obese women presenting with reduced fetal movements compared with women of normal weight [43].

As has been found in other studies, both extremes of parity were shown to be associated with an increased risk of stillbirth [17,44]. In our study grandmultiparous women had the highest risk and Pacific women were more likely to be grandmultiparous than women of other ethnicities. Advanced maternal age can impact upon the relationship between stillbirth and grandmultiparity, however in this study maternal age was included in the adjusted model and the relationship remained. The mechanisms that might underlay the relationship between high parity and stillbirth risk are not yet understood. A speculation by Aliyu and others is that "uterine exhaustion" is reached and the uterus becomes less effective in its nurturing of the fetus [17]. As previous pregnancy complications were not associated with increased risk of stillbirth in this study it is unlikely that the effect of high parity is mediated by adverse outcomes in previous pregnancies.

We found no association between maternal age and stillbirth; this may be due to the study being underpowered for such an association, as previous large population based studies have found associations between both young [45,46] and advanced maternal age [47,48] and stillbirth. Advanced maternal age has also been specifically associated with unexplained stillbirth [49,50], whereas in this study we included all causes of late stillbirth (after exclusion of fetal abnormalities and multiple pregnancies) which may dilute an association between advanced maternal age and unexplained stillbirth in particular. One other possible explanation for our differing findings may be that in two of the three District Health Boards where this study took place, women over 35 years are routinely offered increased antenatal testing in late pregnancy with a low threshold for early induction at term and beyond [51].

A causal relationship between maternal smoking and poor perinatal outcomes is well established [52]. The association between smoking and late stillbirth was significant in univariable analysis but was not in the multivariable analysis, however the aOR is consistent with findings from other larger studies [18,31] and the lack of statistical significance in the current study may be due to insufficient power. Previous studies that have explored the relationship between passive smoking and stillbirth have also shown an increased risk for non-smokers exposed to environmental tobacco smoke [53,54].

This study was powered to detect an odds ratio of 2, with a power of 80% at the 5% level of significance for a risk factor with 20% prevalence, such as obesity or Pacific ethnicity. However it had insufficient power to determine the significance of risk factors with lower prevalence (such as recreational drug use), or that have a smaller strength of association with late stillbirth, such as smoking.

## Conclusion

Pacific ethnicity was not found to be an independent risk factor for late stillbirth. The disparity in stillbirth risk experienced by Pacific women can therefore be attributed to other factors such as obesity and high parity. Further research is required to better understand the mechanisms by which factors such as obesity and high parity are associated with late stillbirth.

## Competing interests

The authors declare that they have no competing interests.

## Authors' contributions

TS participated in the design and coordination of the study, carried out the data collection and drafted the manuscript. JT participated in the design of the study and assisted with statistical analysis and helped to draft the manuscript. EM participated in the conception and design of the study and helped to draft the manuscript. AE participated in the design of the study and helped to edit the manuscript. JZ participated in the design of the study and helped to edit the manuscript. LM participated in the conception and design of the study and helped to draft the manuscript. All authors read and approved the final manuscript.

## Pre-publication history

The pre-publication history for this paper can be accessed here:

http://www.biomedcentral.com/1471-2393/11/3/prepub

## References

[B1] Perinatal and Maternal Mortality Review Committee (PMMRC) Perinatal and maternal mortality in New Zealand 2007 Third report to the Minister of Health 2009 Wellington: Ministry of Health

[B2] Craig E Stewart A Mitchell E Causes of late fetal death in New Zealand 1980-1999 Aust NZ J Obstet Gynaecol 2004 44 441 8 10.1111/j.1479-828X.2004.00284.x 15387867

[B3] New Zealand Health Information Service (NZHIS). Fetal and Infant Deaths 2003 & 2004 2007 Wellington: Ministry of Health

[B4] Confidential Enquiry into Maternal and Child Health (CEMACH) Perinatal Mortality 2007 2009 United Kingdom. London: CEMACH

[B5] Ekeroma A Craig E Stewart A Mantell C Mitchell E Ethnicity and birth outcome: New Zealand trends 1980-2001: Part 3, Pregnancy outcomes for Pacific women Aust N Z J Obstet Gynaecol 2004 44 541 544 10.1111/j.1479-828X.2004.00311.x 15598293

[B6] McCowan LME George-Haddad M Stacey T Thompson JMD Fetal growth restriction and other risk factors for stillbirth in a New Zealand setting Aust N Z J Obstet Gynaecol 2007 47 6 450 6 10.1111/j.1479-828X.2007.00778.x 17991108

[B7] Clarke M Clayton D Mason E MacVicar J Asian mothers' risk factors for perinatal death - the same or different? A 10 year review of Leicestershire perinatal deaths BMJ 1988 297 384 387 10.1136/bmj.297.6645.384 3408977 PMC1834298

[B8] Balchin I Whittaker JC Patel RR Lamont RF Steer PJ Racial variation in the association between gestational age and perinatal mortality: prospective study BMJ 2007 334 7598 833 10.1136/bmj.39132.482025.80 17337455 PMC1853199

[B9] Willinger M Ko CW Reddy UM Racial disparities in stillbirth risk across gestation in the United States Am J Obstet Gynecol 2009 201 5 469.e1 469.e8 10.1016/j.ajog.2009.06.057 PMC2788431 19762004

[B10] Salihu HM Kinniburgh BA Aliyu MH Kirby RS Alexander GR Racial disparity in stillbirth among singleton, twin, and triplet gestations in the United States Obstet Gynecol 2004 104 4 734 740 10.1097/01.AOG.0000139944.15133.e3 15458894

[B11] Canterino J Ananth C Smulian J Harrigan J Vintzileos A Maternal age and risk of fetal death in singleton gestations: USA, 1995-2000 J Matern Fetal Neonatal Med 2004 15 193 197 10.1080/14767050410001668301 15280146

[B12] Fretts R Schmittdiel J McLean F Usher R Goldman M Increased maternal age and the risk of fetal death N Engl J Med 1995 333 15 953 957 10.1056/NEJM199510123331501 7666913

[B13] Nohr EA Bech BH Davies MJ Frydenberg M Henriksen TB Olsen J Prepregnancy obesity and fetal death: a study within the Danish National Birth Cohort Obstet Gynecol 2005 106 2 250 9 10.1097/01.AOG.0000172422.81496.57 16055572

[B14] Cedergren MI Maternal morbid obesity and the risk of adverse pregnancy outcome Obstet Gynecol 2004 103 2 219 24 10.1097/01.AOG.0000107291.46159.00 14754687

[B15] Wisborg K Kesmodel U Henriksen TB Olsen SF Secher NJ Exposure to Tobacco Smoke in Utero and the Risk of Stillbirth and Death in the First Year of Life Am J Epidemiol 2001 154 4 322 327 10.1093/aje/154.4.322 11495855

[B16] Stephansson O Dickman PW Johansson AL Cnattingius S The influence of socioeconomic status on stillbirth risk in Sweden. [see comment] Int J Epidemiol 2001 30 6 1296 301 10.1093/ije/30.6.1296 11821332

[B17] Aliyu MH Salihu HM Keith LG Ehiri JE Islam MA Jolly PE Extreme Parity and the Risk of Stillbirth Obstet Gynecol 2005 106 3 446 453 10.1097/01.AOG.0000165825.65203.69 16135572

[B18] Raymond E Cnattingius S Kiely J Effects of maternal age, parity, and smoking on the risk of stillbirth Br J Obstet Gynaecol 1994 101 301 306 10.1111/j.1471-0528.1994.tb13614.x 8199075

[B19] Stacey T Thompson JM Mitchell EA Ekeroma A Zuccollo J McCowan L The Auckland Stillbirth study, a case control study exploring modifiable risk factors for third trimester stillbirth: methods and rationale Aust N Z J Obstet Gynaecol Article first published online 6 Dec 2010 10.1111/j.1479-828X.2010.01254.x 21299501

[B20] Ministry of Health Ethnicity Data Protocols for the Health and Disability Sector 2004 Ministry of Health New Zealand, Wellington

[B21] WHO Obesity: preventing and managing the global epidemic. report of a WHO Consultation WHO Technical Report Series 894 2000 Geneva: World Health Organisation 11234459

[B22] WHO Expert Consultation Appropriate body-mass index for Asian populations and its implications for policy and intervention strategies Lancet 2004 363 9403 157 63 10.1016/S0140-6736(03)15268-3 14726171

[B23] Swinburn BA Ley SJ Carmichael HE Plank LD Body size and composition in Polynesians Int J Obes Relat Metab Disord 1999 23 11 1178 83 10.1038/sj.ijo.0801053 10578208

[B24] Deurenberg PD-Y M Guricci S Asians are different from caucasians and from each other in their body mass index/body fat per cent relationship Obes Rev 2002 3 3 141 146 10.1046/j.1467-789X.2002.00065.x 12164465

[B25] Razak F Anand SS Shannon H Vuksan V Davis B Jacobs R Teo KK McQueen M Yusuf S Defining obesity cut points in a multiethnic population Circulation 2007 115 16 2111 2118 10.1161/CIRCULATIONAHA.106.635011 17420343

[B26] Salmond C Crampton P Sutton F NZDep91: A New Zealand index of deprivation Aust N Z J Public Health 1998 22 7 835 7 10.1111/j.1467-842X.1998.tb01505.x 9889455

[B27] ARC Council AR The People of the Auckland Region Census series 2006 Auckland Regional Council http://www.arc.govt.nz

[B28] Craig E Mantell C Ekeroma A Stewart A Mitchell E Ethnicity and birth outcome: New Zealand Trends 1980-2001: Part 1. Introduction, Methods, Results and Overview Aust NZ J Obstet Gynaecol 2004 44 441 8 10.1111/j.1479-828X.2004.00284.x 15598291

[B29] Wingate MS Alexander GR Racial and Ethnic Differences in Perinatal Mortality: The Role of Fetal Death Ann Epidemiol 2006 16 6 485 491 10.1016/j.annepidem.2005.04.001 15993623

[B30] Auckland District Health Board National Women's Annual Clinical Report 2009 2010 Auckland: Auckland City Hospital

[B31] Stephansson O Dickman PW Johansson A Cnattingius S Maternal weight, pregnancy weight gain, and the risk of antepartum stillbirth Am J Obstet Gynecol 2001 184 3 463 9 10.1067/mob.2001.109591 11228504

[B32] Salihu HM Dunlop AL Hedayatzadeh M Alio AP Kirby RS Alexander GR Extreme obesity and risk of stillbirth among black and white gravidas. [see comment] Obstet Gynecol 2007 110 3 552 7 10.1097/01.AOG.0000270159.80607.10 17766599

[B33] Ministry of Health A Portrait of Health: Key results of the 2006/7 New Zealand Health Survey 2008 Wellington: Ministry of Health

[B34] Ramos GA Caughey AB The interrelationship between ethnicity and obesity on obstetric outcomes Am J Obstet Gynecol 2005 193 3, Supplement 1 1089 1093 10.1016/j.ajog.2005.06.040 16157117

[B35] Leung TY Leung TN Sahota DS Chan OK Chan LW Fung TY Lau TK Trends in maternal obesity and associated risks of adverse pregnancy outcomes in a population of Chinese women BJOG 2008 115 12 1529 37 10.1111/j.1471-0528.2008.01931.x 19035989

[B36] Smulian J Ananth C Vintzileos A Scorza W Knuppel R Fetal deaths in the United States: influences of the high-risk conditions and implications for management Obset Gynecol 2002 100 6 1183 1189 10.1016/S0029-7844(02)02389-X 12468161

[B37] Sheehan M Jensen M Metabolic complications of obesity Obesity 2000 84 2 363 385 10.1016/s0025-7125(05)70226-1 10793647

[B38] Asfaw A Micronutrient deficiency and the prevalence of mothers' overweight/obesity in Egypt Econ Hum Biol 2007 5 3 471 83 10.1016/j.ehb.2007.03.004 17449338

[B39] Yakoob MY Menezes EV Soomro T Haws RA Darmstadt GL Bhutta ZA Reducing stillbirths: behavioural and nutritional interventions before and during pregnancy BMC Pregnancy Childbirth 2009 9 Suppl 1 S3 10.1186/1471-2393-9-S1-S3 19426466 PMC2679409

[B40] Maasilta P Bachour A Teramo K Polo O Laitinen LA Sleep-Related Disordered Breathing During Pregnancy in Obese Women Chest 2001 120 5 1448 1454 10.1378/chest.120.5.1448 11713118

[B41] Franklin KA Ake Holmgren P Jonsson F Poromaa N Stenlund H Svanborg E Snoring, Pregnancy-Induced Hypertension, and Growth Retardation of the Fetus Chest 2000 117 1 137 141 10.1378/chest.117.1.137 10631211

[B42] Brain KA Thornton JG Sarkar A Johnson AO Obstructive sleep apnoea and fetal death: successful treatment with continuous positive airway pressure BJOG 2001 108 5 543 4 10.1016/S0306-5456(00)00106-6 11368144

[B43] Holm Tveit JV Saastad E Stray-Pedersen B Bordahl PE Froen JF Maternal characteristics and pregnancy outcomes in women presenting with decreased fetal movements in late pregnancy Acta Obstet Gynecol Scand 2009 88 12 1345 51 10.3109/00016340903348375 19878088

[B44] Bai J Wong F Bauman A Mohsin M Parity and pregnancy outcomes Am J Obstet Gynaecol 2002 186 2 274 278 10.1067/mob.2002.119639 11854649

[B45] Wilson RE Alio AP Kirby RS Salihu HM Young maternal age and risk of intrapartum stillbirth Arch Gynecol Obstet 2008 278 3 231 6 10.1007/s00404-007-0557-4 18214510

[B46] de Vienne CM Creveuil C Dreyfus M Does young maternal age increase the risk of adverse obstetric, fetal and neonatal outcomes: A cohort study Eur J Obstet Gynecol Reprod Biol 2009 147 2 151 156 10.1016/j.ejogrb.2009.08.006 19733429

[B47] Salihu HM Wilson RE Alio AP Kirby RS Advanced maternal age and risk of antepartum and intrapartum stillbirth J Obstet Gynaecol Res 2008 34 5 843 50 10.1111/j.1447-0756.2008.00855.x 18834344

[B48] Huang L Sauve R Birkett N Fergusson D van Walraven C Maternal age and risk of stillbirth: a systematic review. [see comment] CMAJ 2008 178 2 165 72 18195290 10.1503/cmaj.070150 PMC2175002

[B49] Froen F Arnestad M Frey K Vege A Saugstad O Stray-Pedersen B Risk factors for sudden interuterine unexplained death: epidemiological characteristics of singleton cases in Oslo, Norway, 1986-1995 Am J Obstet Gynecol 2001 184 4 694 702 10.1067/mob.2001.110697 11262474

[B50] Huang DY Usher RH Kramer MS Yang H Morin L Fretts RC Determinants of unexplained antepartum fetal deaths Obstet Gynecol 2000 95 2 215 21 10.1016/S0029-7844(99)00536-0 10674582

[B51] Fretts R Elkin E Myers E Heffner L Should older women have antepartum testing to prevent unexplained stillbirth? Obstet Gynecol 2004 104 1 56 63 10.1097/01.AOG.0000129237.93777.1a 15229001

[B52] Salihu HM Sharma PP Getahun D Hedayatzadeh M Peters S Kirby RS Alio AP Gaafer-Ahmed H Prenatal tobacco use and risk of stillbirth: a case-control and bidirectional case-crossover study Nicotine Tob Res 2008 10 1 159 66 10.1080/14622200701705431 18188756

[B53] Kharrazi M DeLorenze GN Kaufman FL Eskenazi B Bernert JT Jr Graham S Pearl M Pirkle J Environmental tobacco smoke and pregnancy outcome Epidemiology 2004 15 6 660 70 10.1097/01.ede.0000142137.39619.60 15475714

[B54] Subramoney S d'Espaignet ET Gupta PC Higher risk of stillbirth among lower and middle income women who do not use tobacco, but live with smokers Acta Obstet Gynecol Scand 2010 89 4 572 7 10.3109/00016341003801656 20367432

